# Call for a fairer approach to authorship in publishing biomedical research

**DOI:** 10.1038/s43856-025-00815-9

**Published:** 2025-04-01

**Authors:** Phaik Yeong Cheah, Michael Parker

**Affiliations:** 1 Mahidol Oxford Tropical Medicine Research Unit, Faculty of Tropical Medicine, Mahidol University, Bangkok, Thailand; 2 Centre for Tropical Medicine and Global Health, Nuffield Department of Medicine, University of Oxford, Oxford, UK; 3 Ethox Centre, Nuffield Department of Population Health, University of Oxford, Oxford, UK

**Keywords:** Medical research, Health care

## Abstract

In this Perspective article, we call for a fairer approach to authorship practice in collaborative biomedical research to promote equity and inclusiveness. Current practice does not adequately recognise all contributors involved in different stages of the work and may exacerbate preexisting inequalities. Here, we discuss some key features of contemporary collaborative research practice that complicate authorship decisions. These include the project size, complexity of multidisciplinary team involvement and researchers having varying degrees of expertise and experience. We conclude by making some suggestions to address these concerns.

## Abstract (web summary)

Cheah and Parker discuss how current authorship practice can exacerbate inequalities by excluding some contributors to the work. They call for a fairer approach to determining authors to promote equity and inclusiveness.

## Introduction

Authorship of academic publications confers credit and has important academic, social, and financial benefits. It also implies responsibility, ownership and accountability for published research work^1^. In academia, publications are also frequently used as an important measure of research productivity for both individual researchers and their institutions.

But who should be an author? Despite its importance, not all institutions or research groups have established authorship guidelines. Authorship conventions vary across disciplines, universities and even research groups within the same discipline or university. For biomedical researchers, the most commonly used guidelines are the ‘International Committee of Medical Journal Editors’(ICMJE) guidelines established by ICMJE member journals including the British Medical Journal, the Journal of the American Medical Association, Nature Medicine and the Lancet group of journals^1^. Since 1985, the ICMJE has provided evolving guidance on how authorship should be managed in the complex setting of biomedical science^2^. Many non-ICMJE journals, voluntarily use these recommendations. They are also recommended in international research guidelines such as those of the Council for International Organizations of Medical Sciences (CIOMS)^3^.

The widely influential ICMJE guidelines recommend that authorship be based on the following criteria, all four of which need to have been met to merit authorship^1^: ‘substantial contributions to conception and design, acquisition of data, or analysis and interpretation of data; drafting the article or reviewing it critically for important intellectual content; final approval of the version to be published; and agreement to be accountable for all aspects of the work in ensuring that questions related to the accuracy or integrity of any part of the work are appropriately investigated and resolved.’

The ICMJE does acknowledge the need for some flexibility in the application of these criteria by stating that: ‘The criteria are not intended for use as a means to disqualify colleagues from authorship who otherwise meet authorship criteria by denying them the opportunity to meet criterion 2 or 3. Therefore, all individuals who meet the first criterion should have the opportunity to participate in the review, drafting, and final approval of the manuscript.’ The purpose of this caveat is to address the concern that someone might have made a substantial contribution but have not been given the opportunity to participate in the writing or approval process, thereby disqualifying them from the chance to contribute in all ways that merit authorship. The caveat does not imply any flexibility about the requirements to meet the four criteria but rather requires that everyone who has made an important contribution should be included in the writing and review process if they so wish.

Against this backdrop and acknowledging this caveat, here we discuss some important additional challenges to current authorship practice arising from developments in biomedical research and suggest potential ways forward. Our main focus is how deserving individuals can currently be under-represented in the authorship of publications to which they have made an important contribution. We focus on under-representation in papers that report results of original biomedical research or health-related research (these terms are discussed in the CIOMS 2016 guidelines)^3^, such as clinical trials and qualitative studies.

## Why we need to revisit current authorship practice

Developments in biomedical research practice have begun to put pressure on current approaches to authorship. These developments, discussed in detail below, include the ways in which research teams can be increasingly multidisciplinary and consist of researchers of varying expertise and experience, and the fact that not all intellectually important contributions that come from people who participate in drafting or reviewing academic papers are fluent in English. These issues are further compounded by developments in data sharing regulations. In the following paragraphs, we explore the relevance and impact of each of these in turn.

### Teams are multidisciplinary and consist of researchers of varying expertise and experience

The complexity, scale, and scope of biomedical research continue to expand substantially. During the past few decades an increasing number of large, international, multi-centre clinical trials have been undertaken; multidisciplinary investigations involving interventional studies and qualitative or observational research. These large studies are essential for research to enable sufficient sample size and power to answer important research questions, as well as, to improve the generalisability of the findings. This has several potential advantages, including the ability to bring together different but complementary expertise and experience from many investigators or research areas to address a research topic from different perspectives, and the ability to collect or combine data from many sites or cohorts to generate large data sets to address scientific questions effectively and efficiently. Furthermore, multi-site studies help ensure that participant populations more accurately reflect the intended beneficiaries of the research.

The variety of inputs into such studies can be significant. A team in a large multi-country COVID-19 clinical trial, for example might include project managers, clinicians, statisticians, virologists, data scientists, data managers, genomic experts, ethicists and country or site clinical teams. One of the implications of the need for this cross-disciplinary approach is that inevitably many of the researchers involved will be leading experts in their fields whilst knowing much less about the other areas of research required for the success of the project. They may as a consequence have limited understanding of various facets of the larger project as a whole. Whilst, there will often be a small group of people who understand the project as a whole, for many, perhaps most collaborators with much to offer this will not be the case.

These multi-disciplinary research studies have important implications for many of those involved who have legitimate authorship claims but need to meet the requirements of the ICMJE guidelines. It is, for example, unreasonable for all authors in such studies, despite their expertise in particular aspects of the project and the importance of their contribution, to be held ‘accountable for all aspects of the work in ensuring that questions related to the accuracy or integrity of any part of the work are appropriately investigated and resolved’(ICMJE criterion 4)^1^. The guidelines imply that every single author should be accountable for the work as a whole, but this has the potential to be misinterpreted to suggest that every single author should be accountable for every single aspect of the work. For example, it might be taken to imply that the statistician of a large clinical trial should not only be held accountable for the statistical analyses, but also for the clinical aspects. Whilst it is reasonable to expect an author to be accountable for certain aspects of a publication, which relate to their expertise, a requirement for broader accountability would not reflect the realities of some contemporary team science.

In low- middle-income countries (LMICs), this problem may be exacerbated, for example, where the success of such research requires a strong commitment from the clinician researchers involved at the hospital to drive the study forward and recruit participants. These researchers may often not be seen by project principal investigators (who may be located in a high-income country) to be in a position to meet ICMJE’s 4th criterion. This leads to them being excluded as authors, despite having made an intellectually significant contribution vital to the success of the study. Although, as highlighted above, ICMJE guidelines do emphasise that researchers who meet criterion 1 should not be excluded from meeting criteria 2 and 3, this does not help those researchers who are not in a position to meet criterion 4 because they are not able or judged by others to be able to take responsibility for ‘all aspects of the work’.

The constraints also apply more broadly. Local researchers based in LMICs including early career researchers, full-time clinicians and those who are traditionally considered ‘support staff’ (e.g. data managers, field workers), are disadvantaged in a similar way by current authorship guidelines. In particular, they may not be able to be ‘accountable for all aspects of the work in ensuring that questions related to the accuracy or integrity of any part of the work are appropriately investigated and resolved’ (ICMJE criterion 4) despite their contribution being significant for the overall study success.

### Not all intellectual contributions meriting authorship require drafting or reviewing the paper

In large-scale collaborative research, it is often the case that significant numbers of individuals play pivotal roles contributing intellectual work to a study, but may not be in a position to contribute to the drafting or critical reviewing of papers reporting the study results. This does not imply that such individuals should be excluded from being recognised as authors in scholarly publications. The statement made by ICMJE about the importance of not excluding people who meet criteria 1 and 4 from the opportunity to meet 2 and 3 i.e. to read and review the paper, does not address challenges relating to the fact this kind of contribution may not, even when offered, be feasible for some people who have made other important contributions.

For example, a field worker or interviewer of a qualitative study who has conducted all the interviews in the local language. They do not speak fluent English but have participated fully in discussions in their own language and even led the interpretation of the data. It would be unreasonable for a person in this situation to be expected to write or critically review any text in English. In addition to language difficulties, researchers from LMICs often do not have as much time, experience, training and resources dedicated to academic writing. In these settings, apart from a small number of researchers employed in research institutions, health research is often conducted alongside clinical care and service provision in busy hospitals. Researchers in high-resource settings are more likely to conduct research as an official part of their jobs and are paid to conduct research. The number of full-time equivalent health researchers per million inhabitants is much lower in LMICs than in HICs^4^. This contributes significantly to the under-representation of authors from LMICs when the research is being conducted in their country of base^5^.

Most leading journals are in English, which inevitably disadvantages those for whom English is not their first language. It means non-native speakers including many who have made significant contributions are less able to contribute to the ‘drafting the article or reviewing it critically for important intellectual content’ (ICMJE criterion 2).

These examples illustrate some of the ways in which existing inequalities for example between researchers working in low- and high-resource settings, or junior and senior researchers, and those whose native language is not English can lead to exclusion due to not meeting the authorship requirements set out in ICMJE criterion 2. The playing field is uneven, but this does not mean that the researchers have not provided intellectual content to the project design, analysis or interpretation of the data, as these are typically done through project meetings and discussions rather than actual drafting or reviewing an article in English. It also does not mean that they are undeserving of authorship for their contribution. What this suggests is that in some cases, fulfilling criterion 2 in its current wording, is not always a reasonable requirement for authorship for publication of study results.

### Current practice does not take into account developments in data sharing

In June 2017, the ICMJE published a statement supporting data-sharing practices for randomised controlled trials (RCTs)^6^. It argued that ‘there is an ethical obligation to responsibly share data generated by interventional clinical trials because trial participants have put themselves at risk’ with the aim to ‘maximise the knowledge gained’ from these outstanding studies^6^. Current research guidelines and international research funders such as Wellcome and the Gates Foundation are putting pressure on researchers to share all types of research data, not only RCTs^3^. Arguments to support data sharing include allowing confirmation of the interpretation of results, improving research transparency and trust in science and maximising the utility of the data, with the ultimate aim of improving science and health^7,8^.

As a result of these developments, secondary analyses of individual participant-level data have increased but the volume of data shared remains lower than expected^9^. One of the known challenges of data sharing is the lack of incentives and academic recognition of the investigators who have generated the data of the primary study^10^. Preparing data for sharing involves significant practical and intellectual resources on the part of the primary investigators, which includes data management time, repository management and facilitating a managed access mechanism such as through a data access committee^11^. These primary investigators would only meet criterion 1 (acquisition of data) and hence would currently not fulfil the remaining ICMJE criteria for authorship of any publications arising from the secondary analyses if they are not invited to participate in the secondary analyses.

LMIC researchers have articulated fears over free-riding scientists, especially in those working in HICs, using the data collected by others without adequate recognition^7,8,12^. Although in theory, anyone can conduct the secondary analyses, it is more likely that this will be done by researchers in HICs who have access to more resources, and have data science and writing skills as well as dedicated paid time to conduct research. It has been noted that most data requests come from researchers working in HICs even where the datasets are from LMICs^13^. In such situations, the ICMJE guidelines can be used to exclude primary researchers from the publications arising from the secondary analyses. Unless the primary researchers are invited to participate in the drafting or reviewing of the manuscript, they would not meet criteria for authorship. In earlier versions of the ICMJE guidelines (1985 to 2008), it is stated that ‘participation solely in the collection of data does not justify authorship’^2^. In the 2013 guidelines, a ‘non-author contributors’ section was introduced where it was stated that, “those whose contributions do not justify authorship may be acknowledged…and their contributions should be specified (e.g., served as scientific advisors, critically reviewed the study proposal, collected data…).”^14^ This means that those solely involved in the primary data collection, despite their intellectual contribution to the creation of the original dataset, should not be considered authors in the manuscript arising from the secondary analyses unless they fulfil other criteria set out in the ICMJE guidelines. Although it is arguable that many obstacles to data sharing are not exclusive to those in LMICs, stark resource inequities between researchers in high- and low-resource settings could exacerbate existing inequalities, and in turn, could disincentivize primary data collection and data sharing.

## Suggestions for a way forward

Taken together, the considerations outlined above, many of which arise out of the increasingly international and collaborative nature of contemporary research, and an overly narrow interpretation of the ICMJE guidelines, mean that there are important contributors to successful research who are not currently adequately recognized for their contribution. This has several important implications.

The first and most obvious implication is the lack of recognition. Not recognising someone’s contribution, where they meet reasonable requirements for authorship is to wrong them even if there are no downstream consequences. When the efforts of those who have contributed are not recognized, it represents an inherent injustice, irrespective of any immediate consequences that may or may not arise from this oversight. This issue underscores the importance of giving credit where it is due, and honouring the hard work and dedication of those involved in a research endeavour.

Secondly, the failure to properly reflect contributions in authorship is likely to have negative consequences for those who are excluded due to not meeting the authorship criteria for publication purposes. This may have significant implications for career development. An individual researcher who is denied authorship credit may face a series of detrimental consequences that can hamper their career advancement, thwart future funding opportunities, demotivation, and a sense of being undervalued. Current research practice already disadvantages those working in low-resource settings, early career researchers, those not paid full-time to conduct research (including those who conduct research alongside clinical care), whose native language is not English and those working in supporting roles. Just being a woman is a disadvantage as on average, men across scientific disciplines publish more papers than their female counterparts, and this occurs even when considering obvious mitigating factors^6–9^. Although difficult to prove, current authorship practice exacerbates existing inequalities in research and perpetuates systemic inequities in research, such as career development opportunities and research funding.

Thirdly, publications may be of lower quality due to missing important voices and intellectual input such as voices from researchers from where the study was conducted, viewpoints from LMIC researchers, early career researchers and individuals who played support roles. Results could even be misinterpreted if the local or regional nuances are not factored in the analysis. This raises concerns regarding the credibility of the study and its potential impact on the scientific community and the wider society. Recognising the significance of all contributors is crucial for ensuring comprehensive and credible research outcomes. Emphasising inclusivity and incorporating diverse perspectives enriches the final output of the study, fostering a more robust and relevant body of knowledge.

Fourthly, the current approach may also have wider impacts. It could lead to an undesirable climate for research communities and ultimately be detrimental to the research enterprise as a whole in ways that threaten global health research, team science and collaborations. For example, it may discourage large trials that are crucial to achieve adequate and representative sample size. This will have long term negative consequences to scientific progress. Finally, the sharing of data beyond the primary research team remains low. In many parts of the world, particularly LMICs, despite an ethically sound rationale and growing support for data sharing concerns remain, as described previously^12^. The above-mentioned issues we have outlined suggest a need to reconsider and make amendments to the existing authorship practice. This will require contributions from a range of stakeholders with relevant expertise and experience. We suggest changes along the following lines may be worth considering. First, we would echo the ICMJE guideline that lead researchers have a responsibility to take a proactive role in assisting individuals who meet criterion 1 of the ICMJE guidelines, and who have thereby contributed significant intellectual insights to a study, such as the site principal investigator of a multi-country study to meet all relevant criteria for authorship. In the case of secondary analysis, lead researchers should wherever possible involve key researchers responsible for the collection of the primary dataset in the publication of follow-up analysis. Lead researchers have a responsibility to engage and support less privileged contributing members of the team, finding effective avenues to support early-career researchers, those who face language barriers, and those with limited experience, thus enabling their enhanced contributions. We agree with the ICMJE’s call to ‘encourage collaboration and co-authorship with colleagues in the locations where the research is conducted.’^1^ Mentors and senior authors should go the extra mile in creating authorship pathways for their junior team members, especially researchers from LMICs, and those playing different roles (e.g. data managers, field interviewers, community engagement staff) who may be disadvantaged. This approach not only fosters skill development but also elevates the overall quality of the final research output which benefits the whole research collaboration including lead researchers themselves. Through deliberate inclusion, diverse voices and perspectives are woven into the scholarly narrative, enriching the academic discourse and reinforcing equity in research representation.

Second, we propose an approach to fulfilling criterion 2: ‘Drafting the article or reviewing it critically for important intellectual content’. Intellectual content can take various forms. Some roles, often considered technical may have a substantial intellectual contribution, such as a laboratory technician or data manager. In the case of a lab technician, their work may involve method development, validation, and evaluation. For a data manager, intellectual tasks may include database design and the creation of data collection strategies, as well as data curation.

It is essential to emphasise that researchers who lack proficiency in academic English may not be directly involved in the paper’s writing or reviewing the text in English, and therefore should not be automatically excluded from authorship opportunities. Drafts can be translated into local languages if there are resources, but it is not always pragmatic. Free tools may be used for translating to main languages, but they may not be available for minority languages yet, and if used, they should be used with care. Instead, those who fulfil ICMJE criterion 1, should be invited to engage in meetings and discussions centred around data interpretation, which should enable them to meet this authorship criterion. This approach ensures and provides evidence of their intellectual input without necessitating their involvement in drafting, writing or reviewing the paper in English.

Third, we propose a clarification of ICMJE criterion 4. We argued earlier that there is the potential for criterion 4, ‘Agreement to be accountable for all aspects of the work in ensuring that questions related to the accuracy or integrity of any part of the work are appropriately investigated and resolved’ to be misinterpreted in ways that are overly restrictive. Hence, we propose that further clarification should be provided. Authors should be required to have reasonable confidence in the integrity of the contributions of their co-authors, and all authors should have the general obligation to respond to questions about the work. This does not mean, however, that every author should be accountable for every aspect of the work. A more reasonable expectation is that individual authors should be accountable for the specific aspects of the work that they contributed to and to ensure that questions related to accuracy or integrity are appropriately investigated and resolved.

Finally, we recommend that researchers conducting secondary analyses of data should actively explore inclusion of key primary data producers as authors. We urge researchers conducting secondary analyses to actively involve key members of the team responsible for collecting and curating the primary data, so they can meet criteria 2, 3 and 4. We recognise that this is not always possible but there have been many successful examples of including some involving relatively old datasets. Incorporating insights from those engaged in the primary data collection process significantly enhances the data interpretation phase, enriching the overall analytical perspective.

We would encourage all institutions and research groups to establish a comprehensive data-sharing policy that utilises a managed access system^15^. Such a policy serves to optimise the utilisation of institutional data while safeguarding the interests of the institution, its researchers, and research participants. The policy could include, where appropriate, a small number of researchers from the primary study to be involved in the secondary analysis and be authors on publications arising from any subsequent analysis^11^.

## Conclusion

We have focused primarily on academic-led research collaborations involving large-scale international multidisciplinary teams conducting biomedical research, with a particular focus on LMICs. We are calling for more equitable and inclusive authorship practices. Significant inequities currently exist in the research landscape concerning authorship practices. These inequities particularly disadvantage researchers from low-resource settings, early career researchers, and those who do not write academic English well. Almost by definition, it is difficult to get reliable data on who has been left out as an author. However, we believe the numbers to be significant.

Authorship of research papers is a key means by which significant research contributions in biomedical research are made visible. Authorship is a core metric used in the attribution of credit, decisions about career progression, and respect within the field and across the domains in which those who contribute are employed. For these reasons, the attribution of authorship must be fair and should accurately capture significant research contributions. In this Perspective, we have outlined several obvious reasons why current authorship attribution practices fail to be inclusive and the need for their careful revision. We explore some of the potential ethical and scientific implications of the status quo and suggest some ways of overcoming these challenges.

We recognise that a possible counter-argument to our proposals is that this will lead to unmanageable inflation of author numbers on papers and that this might lead to a devaluing of authorship^16^. This is an empirical claim that may or may not turn out to be true. Authorship decisions need to be justified.

We are not calling for a loosening of authorship requirements, but rather, meaningful contributors to meet the requirements for substantive intellectual contribution, whose input is not captured by the current wording or interpretation of guidelines.

The unwarranted inclusion in authorship lists of those who have not contributed is a different problem, which is not addressed in this Perspective. What is clear is that current practices both fail to adequately recognise those who have contributed substantially to research and limit the potential benefits of diverse perspectives. Science may be missing valuable contributions by not actively working to include diverse voices early in the writing process. Here, we emphasise that authorship implies both credit and accountability^17^. We agree with others in the case of clinical studies, simply being the primary care provider of a research study^18^, as well as merely collecting data should not permit authorship claims^2^. However, existing authorship guidelines may be denying intellectual input to deserving individuals.

We acknowledge that authorships are only one way of recognising contributions. The scope of criteria used to acknowledge impact and academic contributions needs to move beyond the confines of traditional academic publications and related indices such as the *h*-index^19^. Notably, numerous funding bodies and institutions have initiated a shift towards placing greater significance on diverse outputs, encompassing datasets, policy documents, and active engagement with stakeholders and the public. While the tally of publications undeniably retains its significance, it is essential to recognise quantifying publications are not the sole determinant of academic excellence, particularly in the context of research grants and promotions. This evolving perspective highlights the need to embrace a multifaceted approach that encompasses a wide array of contributions, fostering a more comprehensive and inclusive evaluation of scholarly impact and achievement. It is also important to note that there are a range of other ways to recognise contributions (e.g. promotions, salary increase) but those are beyond the scope of this article. Many other aspects of fair collaborations and equitable research partnerships have been detailed elsewhere^20^. Whilst acknowledging and supporting the development of new ways of recognising research contributions, our focus here in this paper has been on the appropriate role and allocation of authorship.

We have not attempted to address authorship challenges of all types of research and collaborations, even though we believe that this issue is likely to be more broadly relevant. We have also not discussed the complementary questions relating to author order, specific authorship challenges in publications arising from ‘big team science’ involving hundreds and thousands of researchers^21^, or industry-sponsored studies which have been discussed elsewhere^22,23^.
