# Author Correction: Call for a fairer approach to authorship in publishing biomedical research

**DOI:** 10.1038/s43856-025-00857-z

**Published:** 2025-04-24

**Authors:** Phaik Yeong Cheah, Michael Parker

**Affiliations:** 1 Mahidol Oxford Tropical Medicine Research Unit, Faculty of Tropical Medicine, Mahidol University, Bangkok, Thailand; 2 Centre for Tropical Medicine and Global Health, Nuffield Department of Medicine, University of Oxford, Oxford, UK; 3 Ethox Centre, Nuffield Department of Population Health, University of Oxford, Oxford, UK

**Keywords:** Medical research, Health care

Correction to: *Communications Medicine* 10.1038/s43856-025-00815-9, published online 01 April 2025

In the version of this article initially published, there was a typo in the Conclusion section. In the sentence now reading, “These inequities particularly disadvantage researchers from low-resource settings, early career researchers, and those who do not write academic English well,” the word “not” was missing. This has now been amended in the HTML and PDF versions of the article.

